# Serum CA19-9 Level Associated with Metabolic Control and Pancreatic Beta Cell Function in Diabetic Patients

**DOI:** 10.1155/2012/745189

**Published:** 2012-06-12

**Authors:** Haoyong Yu, Ruixia Li, Lei Zhang, Haibing Chen, Yuqian Bao, Weiping Jia

**Affiliations:** ^1^School of Medicine, Shanghai Jiao Tong University, Shanghai 200233, China; ^2^Department of Endocrinology and Metabolism, Shanghai Jiao Tong University Affiliated Sixth People's Hospital, Shanghai Diabetes Institute, Shanghai Clinical Center of Diabetes, Shanghai Key Laboratory of Diabetes Mellitus, Shanghai 200233, China; ^3^Department of medicine, Soochow University, Soochow 215006, China

## Abstract

CA19-9 is a tumor-associated antigen. It is also a marker of pancreatic tissue damage that might be caused by diabetes. Long-term poor glycemic control may lead to pancreatic beta cell dysfunction which is reflected by elevated serum CA19-9 level. Intracellular cholesterol accumulation leads to islet dysfunction and impaired insulin secretion which provide a new lipotoxic model. This study firstly found total cholesterol was one of the independent contributors to CA19-9. Elevated serum CA19-9 level in diabetic patients may indicate further investigations of glycemic control, pancreatic beta cell function, and total cholesterol level.

## 1. Introduction

CA19-9 is a tumor-associated antigen that was originally defined by a monoclonal antibody produced by a hybridoma prepared from murine spleen cells immunized with a human colorectal cancer cell line. Although increased serum CA19-9 level is known to be associated with pancreatic cancer. In particular, it has been also shown to increase in many malignant diseases such as upper gastrointestinal tract, ovarian, and hepatocellular and colorectal cancer. In addition, various studies have reported increased serum CA19-9 levels in benign diseases such as inflammatory conditions of the hepatobiliary system, thyroid disease [1], acute and chronic pancreatitis [2], diabetes mellitus [3] (DM), and interstitial pulmonary disease [4].

CA19-9 is used in the diagnosis of pancreatic cancer, but it is also a marker of pancreatic tissue damage that might be caused by diabetes. Benhamou et al. [5] investigated the relationship between the CA19-9 and metabolic control of diabetes in 51 adult patients. They concluded that CA19-9 in diabetic patients is raised in acute metabolic situations, which correlated very well with blood glucose concentration. It was suggested that glucose toxicity may play a role in high serum CA19-9 levels in these patients. Gul et al. [6] showed that serum CA19-9 level was related to microvascular complications in type 2 DM patients.

The aim of this study was to evaluate serum CA19-9 levels in patients with DM in comparison with age- and sex-matched control subjects. In addition, we aimed to find out whether serum CA19-9 level was related with metabolic control and pancreas pancreatic beta cell function in these subjects.

## 2. Research Design and Methods

### 2.1. Study Population

71 type 1 DM, 866 type 2 DM patients, and 122 healthy volunteers who examined and treated in our outpatient clinic and inpatient department were enrolled in this cross-sectional study. The local ethical committee approval was obtained. Patients with malignant disease, with history of chemotherapy or radiotherapy, and with acute or chronic pancreatitis were excluded. Patients with diabetes who have any coexistent disease related to high CA19-9 levels were also excluded. CA19-9 levels were measured in all subjects. Cases with high CA19-9 levels were evaluated with abdominal ultrasonography and CT imaging. Upper gastrointestinal endoscopy and colonoscopy were performed when needed. Duration of diabetes was calculated by years. Heights and weights of the participants were measured, and their body mass indexes (BMI) [weight (kg)/square of height (m^2^)] were calculated.

### 2.2. Laboratory Tests

Plasma glucose was assayed by glucose oxidase method. Serum C peptide concentration was measured by radioimmunoassay (RIA) (Linco Research, United States). HbA1c was determined by high-performance liquid chromatography (Bio-Rad Inc., Hercules, USA). GA was measured by enzymatic method (LUCICA GA-L, Asahi KASEI, Tokyo). Alanine aminotransferase (ALT) was measured by UV method. Alanine aminotransferase (AST) was measured by Szasz-Persijn method. Serum triglyceride (TG), TC, high-density lipoprotein cholesterol (HDL-c), and low-density lipoprotein cholesterol (LDL-c) were measured by enzymatic procedures using an autoanalyzer (Hitachi 7600-020, automatic analyzer, Japan). Serum CA19-9 level was measured using chemiluminescence method and access GI monitor kit (Siemens Immulite 2000, Siemens Healthcare Diagnostics; and Immulite 2000, Beckman Coulter, Brea, CA). Normal ranges for serum CA19-9 level were 0 to 35 U/mL, and the levels above higher range were accepted as abnormal.

### 2.3. Statistical Methods

All analyses were performed with Statistical Package for Social Sciences 11.0 software (SPSS, Chicago, USA). Data were expressed as mean ± SD except skewed variable which was presented as medium (interquartile range 25%–75%), and the data that were not normally distributed were logarithmically transformed before analysis. Clinical characteristics were compared among the three groups using one-way ANOVA test, and several variables without data of control group were compared with independent samples *t* test. The Pearson and Spearman correlation coefficients were calculated to assess the strength of the correlation of CA19-9 and parameters of glucose and lipid metabolism. The ΔCP represents the difference between the value of CP 120 min and CP 0 min, which regarded as an important indicator of pancreatic beta cell function because many diabetic patients were treated with exogenous insulin. Multiple stepwise regression analysis was performed to determine the associations between serum CA19-9 and metabolic parameters. The variables selected to enter into stepwise regression were those that correlated significantly with serum CA19-9 (after correlation analysis). All reported *P* values were two-tailed and *P* < 0.05 were considered statistically significant.

## 3. Results

### 3.1. Characteristics of Subjects

The general characteristics and clinical parameters of the cross-sectional study are summarized in Table 1. Age, duration of diabetes, TG, HDL-c, blood urea nitrogen (BUN), FPG, 2hPG, HbA1c, GA, CP0, CP120, ΔCP, and CA19-9 level differed significantly among the three groups (*P* < 0.01). People with type 1 and type 2 diabetes had significantly higher FPG, 2hFPG, HbA1c, ΔCP, and CA19-9 level than control group (*P* < 0.01). In addition, there was significant difference between two of the three groups in FPG, CP0, and ΔCP (*P* < 0.01).

### 3.2. CA19-9 Value Quartile

As is shown in Table 2, the subjects were divided into 4 quartiles on the basis of CA19-9 values. Compared with lower quartile group, the upper quartile group had significant higher FPG, 2hFPG, HbA1c, GA and lower CP0, CP120, and ΔCP (*P* < 0.01). Among all of parameters, HbA1c, GA, and ΔCP had statistic significance in every two groups.

### 3.3. Correlation Analysis in Groups

In whole participants the correlation analysis (Table 3) showed that serum CA19-9 was positively correlated with TC (*r* = 0.129, *P* < 0.001) FPG (*r* = 0.309, *P* < 0.001), 2hPG (*r* = 0.284, *P* < 0.001), HbA1c (*r* = 0.486, *P* < 0.001), and GA (*r* = 0.389, *P* < 0.001) and was negatively correlated with CP0 (*r* = −0.229, *P* < 0.001), CP120 (*r* = −0.365, *P* < 0.001), and ΔCP (*r* = −0.359, *P* < 0.01).

### 3.4. Multiple Stepwise Regression Analysis in Groups

To further determine which variables were independently associated with serum CA19-9, multiple stepwise regression analysis was performed (Table 4). We selected the parameters which significantly correlated with serum CA19-9 level showed in Table 3 as independent, serum CA19-9 levels as dependent. As a result, HbA1c, type of diabetes, TC, and ΔCP were independently associated with serum CA19-9 levels.

## 4. Discussion

This is the first study which demonstrated that increased serum CA19-9 level significantly correlated with serum total cholesterol and pancreatic beta cell function in diabetic patients.

CA19-9 is a tumor marker mainly used for the diagnosis of pancreatic cancer. However, it is well known that high serum CA19-9 levels can also be found in various diseases, such as nonmalignant obstructive jaundice, thyroid disease, and ovarian diseases. In limited numbers of studies with small sample sizes, patients with diabetes were shown to have increased CA19-9 levels compared with control groups [7, 8]. In our study, we also demonstrated that diabetic patients have increased serum CA19-9 levels than control. Interestingly, we further found that CA19-9 levels in type 1 diabetes were higher than in type 2 diabetes, although there was no significant difference in HbA1c between the two groups.

HbA1c is a marker of chronic glucose toxicity. Significant correlation was also defined between serum CA19-9 levels and HbA1c. In a previous study [9], it was shown that patients with poor glucose control had the highest serum CA19-9 levels. Long-term poorly glycemic control can lead to chronic oxidative stress, which is a central mechanism for glucose toxicity. Our study also demonstrated the positive correlation between the CA19-9 and HbA1c levels. According to CA19-9 value quartile, the upper quartile group had significant higher HbA1c, GA and lower CP0, CP120, ΔCP than the lower quartile group. The multiple stepwise regression analysis also showed that HbA1c was one of the major independent contributors to CA19-9. These results extend those from previous studies and provide additional evidence that long-term poor glycemic control may lead to pancreatic beta cell dysfunction which is reflected by elevated serum CA19-9 level.

The mechanism of increased serum CA19-9 levels in diabetic patients remains unclear. One of them is that the rise of serum CA19-9 level only reflects cellular dysfunction. The lack of insulin could result in a pancreatic exocrine deficiency and release of CA19-9 by ductal cells [10]. Therefore, the increase of serum CA19-9 level might parallel the intensity of cellular functional disorders. Many early studies on pancreatic function in diabetes demonstrated that pancreatic exocrine insufficiency is present in a considerable percentage of patients with diabetes. Autopsy studies and studies on pancreas histology showed marked changes in the exocrine gland in patients with diabetes mellitus as compared to the nondiabetic controls [11]. Blumenthal et al. reported signs of chronic inflammatory changes of the exocrine pancreas in 11.2% of patients with diabetes mellitus as compared to 5.3% in nondiabetic patients [12]. Therefore, the elevated serum CA19-9 level in diabetic patients might be explained by exocrine damaged. In our study, we firstly found that serum CA19-9 level was negatively correlated with ΔC-peptide which reflected the pancreatic beta cell function. Type 1 diabetes is a chronic progressive autoimmune disease, which leads to the loss of pancreatic beta cell. The level of serum CA19-9 in type 1 diabetes was higher than type 2 diabetes. Multiple stepwise regression analysis showed that type 1 diabetes is an independent contributor to CA19-9. These results supported that increased serum CA19-9 levels may well be a biomarker of damaged pancreatic beta cell function.

Diabetes is often accompanied by abnormal blood lipid and lipoprotein levels, but most studies on the link between dyslipidemia and diabetes have focused on TG and free fatty acids (FFAs). More recently, the accumulating data suggested that cholesterol homeostasis is a major regulator of pancreatic beta cell function [13]. Intracellular cholesterol accumulation leads to islet dysfunction and impaired insulin secretion which provide a new lipotoxic model and a potential link of disturbed cholesterol metabolism to impairment of pancreatic beta cell function [14]. Hao et al. [15] indicated that excess cellular cholesterol plays a direct role in islet pancreatic beta cell dysfunction and may be a key factor underlying the progression of type 2 diabetes. Using different animal models, they showed that elevated serum cholesterol leads to increased cholesterol in pancreatic islets. More importantly, islet cholesterol levels directly and significantly impact the extent of glucose-stimulated insulin secretion, independent of FFAs levels. In our literature, multiple stepwise regression analysis showed that TC was one of the independent contributors to CA19-9. This result further indicates that the increased serum TC level may be associated with the decrease of pancreatic beta cell function. It has great implications that the regulation of cholesterol level may be a potential target for therapeutic intervention aimed at preserving or improving pancreatic beta cell function.

The elevated serum CA19-9 level in diabetic patients may indicate further investigations of glycemic control, pancreatic beta cell function, and TC level. One limitation of the present study should be noted that it was a cross-sectional study. A long-term follow-up study of these subjects should be undertaken to further determine the correlation of serum CA19-9 level with pancreatic beta cell function and TC level.

## Figures and Tables

**Table 1:** Demographic and clinical characteristics of study subjects.

Variables	Control (*n* = 122)	T1DM (*n* = 71)	T2DM (*n* = 866)	*P* value
Gender (M/F)	64/58	34/37	507/359	—
Age (y)	47.78 ± 11.23	51.68 ± 17.84^∗##^	60.45 ± 11.97^∗∗^	<0.001
Duration (y)	—	5.00 (1.00–8.00)^##^	8.00 (3.88–13.00)	0.008
ALT (U/L)	18.2 (13.00–26.75)	17.00 (11.00–27.50)	18.0 (13.00–29.00)	0.722
AST (U/L)	21.0 (17.00–25.00)	18.00 (15.00–25.00)	19.0 (15.00–24.00)	0.332
TC (mmol/L)	4.84 ± 0.85	4.60 ± 1.12	4.75 ± 1.20	0.401
TG (mmol/L)	1.20 (0.87–1.79)	0.90 (0.71–1.33)^∗##^	1.52 (1.06–2.20)^∗∗^	<0.001
HDL (mmol/L)	1.35 ± 0.32	1.40 ± 0.42^##^	1.11 ± 0.35^∗∗^	<0.001
LDL (mmol/L)	3.18 ± 0.87	2.97 ± 1.12	3.17 ± 0.98	0.256
BUN (mmol/L)	4.83 ± 1.21	6.04 ± 2.83^∗∗^	5.82 ± 2.47^∗∗^	<0.001
Cr (*μ*mol/L)	67.00 (58.00–78.00)	65.50 (51.25–79.75)	66.00 (54.00–80.00)	0.855
FPG (mmol/L)	5.08 ± 0.34	9.60 ± 4.21^∗∗##^	8.48 ± 3.20^∗∗^	<0.001
2hPG (mmol/L)	6.06 ± 1.09	14.09 ± 6.17^∗∗^	14.24 ± 4.76^∗∗^	<0.001
HbA1c (%)	5.43 ± 0.33	9.81 ± 2.82^∗∗^	9.34 ± 2.33^∗∗^	<0.001
GA (%)	—	29.30 ± 8.82^##^	25.94 ± 8.41	0.001
CP0 (ng/mL)	6.84 (5.08–9.85)	0.25 (0.05–0.80)^∗∗##^	1.62 (1.04–2.31)^∗∗^	<0.001
CP120 (ng/mL)	36.80 (22.44–57.97)	0.40 (0.48–1.42)^∗∗^	3.37 (2.05–5.29)^∗∗^	<0.001
ΔCP (ng/mL)	28.84 (16.43–52.42)	0.08 (0.00–0.62)^∗∗##^	1.59 (0.77–2.77)^∗∗^	<0.001
CA19-9 (KU/L)	4.69 (2.66–9.65)	18.59 (11.68–39.28)^∗∗##^	12.07 (6.72–21.57)^∗∗^	<0.001

Data represent means ± S.D. or median (interquartile range), ^∗^ *P* < 0.05,  ^∗∗^ *P* < 0.01 versus control group; ^#^ *P* < 0.05, ^##^ *P* < 0.01 versus T2DM group.

ALT, aspartate aminotransferase; AST, alanine aminotransferase; BUN, blood urea nitrogen; Cr, creatinine; FPG, fasting plasma glucose; 2hPG, 2h plasma glucose; HbA1c, glycated hemoglobin A1C; GA, glycated serum albumin; TG, total triglycerides; TC, total cholesterol; HDL, high-density lipoprotein; LDL, low-density lipoprotein; CP0, C Peptide of 0 min; CP120, C Peptide of 120 min; ΔCP, D value of C Peptide of 120 min minus C Peptide of 0 min.

**Table 2:** Basic clinical and biochemical characteristics by quartiles of CA19-9.

Variables	CA19-9 quartile				*P* value
	1 (Lowest)	2	3	4 (Highest)
Age (y)	56.64 ± 12.198	60.1 ± 12.652^∗^	58.75 ± 13.534	58.21 ± 13.806	0.023
Duration (y)	8.0 (3.0–12.0)	9.0 (4.0–12.5)	8.0 (3.0–13.0)	8.0 (3.0–13.0)	0.327
ALT (U/L)	17.00 (13.00–26.00)	19.00 (13.75–30.00)	17.00 (13.00–75.00)	19.00 (12.00–29.00)	0.398
AST (U/L)	19.00 (15.00–24.00)	19.00 (16.00–24.75)	18.50 (16.00–24.00)	19.00 (16.00–25.00)	0.508
TC (mmol/L)	4.66 ± 0.935	4.62 ± 1.054	4.75 ± 1.201	4.99 ± 1.367^∗∗##▲^	0.001
TG (mmol/L)	1.45 (0.99–2.08)	1.43 (1.04–2.04)	1.47 (0.93–2.07)	1.44 (0.97–2.21)	0.739
HDL (mmol/L)	1.18 ± 0.428	1.12 ± 0.326^∗^	1.15 ± 0.328	1.18 ± 0.368	0.167
LDL (mmol/L)	3.11 ± 0.849	3.10 ± 0.914	3.16 ± 1.032	3.27 ± 1.086	0.168
BUN (mmol/L)	5.37 ± 1.615	5.84 ± 2.517	5.75 ± 2.340	5.91 ± 2.931	0.056
Cr (*μ*mol/L)	67.00 (57.00–78.25)	68.00 (57.00–81.00)	64.00 (54.00–77.00)	63.00 (51.75–80.50)	0.363
FPG (mmol/L)	6.84 ± 2.367	7.93 ± 2.752^∗∗^	8.73 ± 3.263^∗∗#^	9.15 ± 4.116^∗∗##^	<0.001
2hPG (mmol/L)	10.77 ± 4.70	13.72 ± 4.812^∗∗^	13.95 ± 5.285^∗∗^	14.72 ± 5.471^∗∗^	<0.001
HbA1c (%)	7.36 ± 1.870	8.41 ± 1.945^∗∗^	9.43 ± 2.444^∗∗##^	10.49 ± 2.768^∗∗##▲▲^	<0.001
GA (%)	21.89 ± 5.874	24.30 ± 6.819^∗^	27.18 ± 8.455^∗∗##^	30.29 ± 9.525^∗∗##▲▲^	<0.001
CP0 (ng/mL)	2.195 (1.383–4.228)	1.80 (1.18–2.695)^∗^	1.705 (1.04–2.553)^∗∗^	1.29 (0.64–2.03)^∗∗#^	<0.001
CP120 (ng/mL)	5.32 (2.85–15.05)	4.21 (2.53–5.82)^∗∗^	3.27 (1.76–5.44)^∗∗#^	2.38 (1.31–4.15)^∗∗#^	<0.001
ΔCP (ng/mL)	2.76 (1.34–9.388)	2.09 (0.94–3.41)^∗∗^	1.55 (0.53–2.77)^∗∗#^	1.00 (0.41–2.06)^∗∗#▲^	<0.001

Data represent means ± S.D. or median (interquartile range), ^∗^ *P* < 0.05, ^∗∗^ *P* < 0.01 versus group 1; ^#^ *P* < 0.05,  ^##^ *P* < 0.01 versus proup 2; ^▲^ *P* < 0.05,  ^▲▲^ *P* < 0.01 versus proup 3.

**Table 3:** Correlation analysis of serum CA19-9 with variables as follows.

Variables	Serum CA19-9	
	*r*	*P*
Age	0.045	0.142
Duration	0.004	0.904
ALT	0.029	0.35
AST	0.038	0.217
TC	0.129^∗∗^	<0.001
TG	0.048	0.125
HDL	−0.002	0.961
LDL	0.068^∗^	0.028
BUN	0.057	0.07
Cr	−0.064^∗^	0.041
FPG	0.309^∗∗^	<0.001
2hFPG	0.284^∗∗^	<0.001
HbA1c	0.486^∗∗^	<0.001
GA	0.389^∗∗^	<0.001
CP0	−0.229^∗∗^	<0.001
CP120	−0.365^∗∗^	<0.001
ΔCP	−0.359^∗∗^	<0.001

^∗^ *P* < 0.05, ^∗∗^ *P* < 0.01.

**Table 4:** Multiple stepwise regression analysis showing variables independently associated with serum CA19-9.

Independent variables enter the model	*β*	S.E.M	Standardized *β*	*t*	*P*	95% CI for *β* (lower limit-upper limit)
HbA1c	0.068	0.005	0.433	13.223	<0.001	0.058 to 0.078
Type of diabetes	−0.139	0.043	−0.099	−3.235	0.001	−0.223 to −0.055
TC	0.028	0.009	0.091	3.02	0.003	0.010 to 0.046
ΔCP	−0.019	0.007	−0.084	−2.547	0.011	−0.033 to −0.004

The parameters which significantly correlated with serum CA19-9 level showed in Table 3 were selected to enter into the model.
